# The suppression effect of SCH-79797 on *Streptococcus mutans* biofilm formation

**DOI:** 10.1080/20002297.2022.2061113

**Published:** 2022-04-18

**Authors:** Lingjun Zhang, Yan Shen, Lili Qiu, Fangzheng Yu, Xiangyu Hu, Min Wang, Yan Sun, Yihuai Pan, Keke Zhang

**Affiliations:** School and Hospital of Stomatology, Wenzhou Medical University, Wenzhou, Zhejiang, China

**Keywords:** *Streptococcus mutans*, SCH-79797, biofilms, dental caries, cariogenic virulence

## Abstract

**Background:**

SCH-79797 was recently shown to be a broad-spectrum antibacterial agent with a dual-bactericidal mechanism. However, its anti-biofilm effect remains unknown.

**Purpose:**

To investigate the effect of SCH-79797 on the biofilm formation of the cariogenic *Streptococcus mutans*

**Methods and Results:**

Crystal violet staining, colony forming units count and MTT assays (for cell metabolic activity) revealed that *S. mutans* biofilm formation was significantly suppressed. In addition, virulence factors, including extracellular polysaccharides (investigated by bacterial/exopolysaccharide staining and the anthrone method) and acid production (investigated by lactic acid and supernatant pH detection) were also inhibited significantly. Moreover, the biofilm inhibitory effect of SCH-79797 was mediated through its repression of bacterial growth and not by a bactericidal effect, which was verified by growth curve and bacterial live/ dead staining, respectively. Quantitative real-time PCR results disclosed that SCH-79797 affected bacterial acid production and tolerance, polysaccharide synthesis and remodeling, biofilm formation and quorum sensing-related gene expression. In addition, SCH-79797 showed good biocompatibility as determined by cytotoxicity assays.

**Conclusion:**

SCH-79797 had an anti-biofilm effect and showed application prospects in the control of dental caries.

## Introduction

Dental caries is one of the most prevalent, non-communicable infectious disease worldwide [1]. According to the latest global disease research statistics, approximately 34.1% of permanent teeth and 7.8% of deciduous teeth worldwide are affected by caries [2]. Therefore, the prevention and treatment of dental caries is a global public health challenge. *Streptococcus mutans* is one of the main cariogenic agents of dental caries. It is closely related to tooth decay and reducing or eradicating *S. mutans* can significantly reduce the occurrence of dental caries [3]. The cariogenic virulence factors of *S. mutans* include acid production, acid tolerance, and biofilm formation [4]. The acid production of *S. mutans* can be regulated by lactate dehydrogenase (LDH), which is encoded by *ldh*. During carbohydrate metabolism, LDH promotes the synthesis of lactic acid, which dissolves the hard tissue of the tooth and cause caries over time [5]. The acid tolerance of *S. mutans* is mediated through the activity of F1F0-ATPase, which acts as a pump to expel protons from the cell to maintain the internal pH; thus, conferring a survival advantage under conditions of acid stress [6]. As an important producer of extracellular polymeric substances within dental plague, *S. mutans* is able to synthesize intracellular and extracellular polysaccharides (EPS) through glucosyltransferases (Gtfs) [4]. EPS can be divided into water-insoluble glucan (WIG) which plays an important role in the construction of the three-dimensional scaffold network of dental biofilms and aids the binding of bacteria on the tooth surface, and water-soluble glucan (WSG). The α-1,6-glycosidic bond of WSG can be decomposed by water-soluble glucanohydrolase; this process supplies energy for the bacterium [7]. Robust mature biofilm with three-dimensional architecture presented the greatest resistance to antibiotics and its resistance could even reach 100–1,000 times that of planktonic bacteria. This protects the bacteria within the biofilm from destruction, making the prevention of dental caries a challenging prospect [8].

At present, non-specific methods, such as toothbrushes and dental floss, are generally used to remove the cariogenic biofilms on the tooth surface [9]. In addition, various antibacterial agents act on dental biofilms. These are primarily divided into the following categories: antibiotics, antimicrobial enzymes, antimicrobial peptides, cationic compounds, metal and metal oxides, other non-cationic compounds, natural products, amino acids and antioxidants [10]. Unfortunately, various clinically used antibacterial agents display side effects such as gastrointestinal reactions, mental addiction, or tooth discoloration [11]. In addition, usually there is only one bacterial target for a lot of antibacterial agents which could result in drug resistance more easily. For example, aminoglycosides are the most commonly used antibiotics that inhibit bacterial protein synthesis to achieve antibacterial effects. It been confirmed that *S. mutans* can develop high levels of resistance to it [12,13]. Chlorhexidine, which is the gold standard for controlling dental plaque clinically, acts on bacterial cell walls and induces strain resistance in *S. mutans* [14]. The imperceptible low-frequency resistance of the drug may be due to its multiple different targets [15]. Therefore, antibacterial agents with multiple targets to avoid bacterial resistance are attracting more attention.

SCH-79797, once considered to be an antagonist of protease activated receptor 1, has a certain therapeutic effect on cardiac ischemia, reperfusion arrhythmias and amyotrophic lateral sclerosis [16,17]. Recently, SCH-79797 was identified as a broad-spectrum antibiotic that significantly hindered the growth of various of Gram-negative and Gram-positive pathogens, such as *Escherichia coli, Neisseria gonorrhoeae, Acinetobacter baumannii, Enterococcus faecalis* and *Staphylococcus aureus*, and exhibited strong and rapid bactericidal activity *in vitro* [15]. Moreover, SCH-79797 exceeded combination therapy in the treatment of clinical isolates of methicillin-resistant *S. aureus* (MRSA) persisters [15]. Besides, its antibacterial efficacy *in vivo* was confirmed in a case of an animal host infection in which a wax worm was infected by *A. baumannii* [15]. Owing to its simultaneous targeting of folate metabolism and membrane integrity, SCH-79797 exhibited undetectable drug resistance [15]. Therefore, SCH-79797 was a promising candidate antibiotic. There have been no reports to date on the effect of SCH-79797 on biofilms, specifically those of the cariogenic bacterium *S. mutans*. The present study aimed to explore the antibacterial activity of SCH-79797 against *S. mutans* biofilms.

## Materials and methods

### Bacterial strains and growth conditions

*S. mutans* UA159 was routinely inoculated into brain heart infusion (BHI; Oxoid, Basingstoke, UK) at 37°C and 5% CO_2_ (v/v). For the growth curve, 200 μl of BHI containing 10^6^ CFU/ml *S. mutans* and different concentrations of SCH-79797 were cultured in 96-well plates. BHI (2 ml) with 1% sucrose (m/v; BHIS), 10^6^ CFU/ml *S. mutans* and different concentrations of SCH-79797 were used for biofilm formation in 24-well plates with pre-placed circular glass sheets except for the crystal violet assay, in which 96-well plates and 200 μl culture volume were chosen. SCH-79797, commercially obtained from Santa Cruz Biotechnology (Dallas, TX), was dissolved in DMSO at 25 mM as stock solution. DMSO (1%) was used as DMSO control, whereas the group without SCH-79797 was used as blank control. For all bacteria related assays including biofilm formation and growth, SCH-79797 was added at the beginning and incubated for 24 h.

### Crystal violet assay

Crystal violet staining was used to estimate the biofilms biomass after biofilm formation [18]. After fixing the biofilms in 96-well plates with methanol for 15 min, the supernatant was discarded and the biofilms were air-dried. Then, 100 μl of 0.1% crystal violet was added into each well and incubated for 20 min. After the crystal violet was aspirated, the biofilms were cleaned with sterile deionized water, then air-dried. A stereo microscope (Nikon SMZ800, Nikon, Tokyo, Japan) was used to examine the stained biofilms. For quantitative analysis, the crystal violet stain in the biofilm was dissolved in 33% acetic acid, and its optical density (OD) was measured using a microplate reader (SpectraMax M5, Molecular Devices, San Jose, CA) at an absorbance of 590 nm.

### Biofilm colony forming unit (CFU) count

The CFU count was used to determine the number of colonies in *S. mutans* biofilm after 24 h biofilm formation [19]. After removing planktonic bacteria, the mechanically scraped biofilm was suspended in PBS, mixed drastically and diluted in sequence, and then spread on the BHI plates for growth. After incubation at 37°C under 5% CO_2_ for 48 h the colonies were counted.

### MTT assay

The effect of SCH-79797 on the viability of *S. mutans* biofilms was evaluated by using MTT assay after 24 h biofilm formation [20]. After washing with PBS, the biofilms in the 24-well plates were transferred to a new 24-well plate. MTT (1 ml; 0.5 mg/ml) was added to each well and incubated in 5% CO_2_ at 37°C. After 1 h, the MTT was replaced with 1 ml DMSO, and the incubation continued for 20 min. The absorbance of 200 μl of the above solution was measured at 540 nm.

### Water-insoluble glucan measurement

After 24 h biofilm formation, the water-insoluble EPS of *S. mutans* biofilms was determined using the anthrone method [21]. *S. mutans* biofilms treated with SCH-79797 were obtained according to the above method. Biofilms were collected, washed with PBS, and resuspended in 0.4 M NaOH. After centrifugation, 100 μl of the supernatant was mixed with 300 μl of anthrone reagent and incubated at 95°C for 6 min. Absorbance at 625 nm of the 200 μl solution was read (SpectraMax M5, Molecular Devices), and the corresponding concentration was calculated based on a standard curve of dextran.

### Bacteria/extracellular polysaccharide staining

The bacteria/EPS with biofilms were stained as previously described [22]. In short, 2.5 μM Alexa Fluor 647-dextran conjugate (Molecular Probes, Invitrogen Corp., Carlsbad, CA) together with different concentrations of SCH-79797 were added to each well and incubated with biofilms for 24 h. Next, 2.5 μM SYTO 9 (Molecular Probes, Invitrogen Corp) was used to stain *S. mutans* within the biofilms after biofilm formation. The biofilms were then examined under a laser confocal microscope (Nikon A1, Nikon Corporation, Japan) using a 60 × objective lens. *S. mutans* was stained green by SYTO 9 (maximum excitation/emission is 480/500 nm), and EPS was stained red by Alexa Fluor 647-dextran conjugate (maximum excitation/emission is 650/668 nm) in three-dimensional reconstructed biofilms.

### Lactic acid and supernatant pH measurement

Lactic acid and supernatant pH measurement reflect the acid-producing ability of *S. mutans* biofilms [23]. After biofilm formation, Cysteine Peptone Water (CPW) was used to wash the biofilms, which were then transferred to a new 24-well plate. Buffered Peptone Water (BPW; 1.5 ml) containing 0.2% sucrose was added to each well and incubated for 3 h under 5% CO_2_ at 37°C to produce acid. LDH was used to quantify the lactate concentration in the BPW solution. The absorbance was monitored at 340 nm, and the lactic acid content was calculated based on the standard curve generated by a lactic acid standard. The pH of the supernatant after 24 h incubation of *S. mutans* biofilms was measured using a pH meter (Mettler Toledo Instruments Co. Ltd., Shanghai, China).

### Growth curve

A growth curve was drawn to detect the effect of SCH-79797 on the growth of *S. mutans* [24]. *S. mutans* cultured overnight was incubated with different concentrations of SCH-79797 or 1% DMSO for 24 h, and the final concentration of bacteria was adjusted to 10^6^ CFU/ml in each well of a 96-well plate (200 μl final volume per well). The OD of each well was measured every 2 h at 600 nm using a microplate reader (SpectraMax M5).

### Live/dead bacteriaviability assay

Bacteria viability within the biofilms was assessed using the BacLight live/dead bacterial viability kit (Molecular Probes, Eugene, OR) [25]. The 24 h biofilms cultured with SCH-79797 or DMSO were stained with 2.5 μM SYTO 9 and 2.5 μM propidium iodide for 30 min. A confocal laser scanning microscope (Nikon A1, Nikon Corporation, Japan) was used to examine the bacteria; live bacteria were stained green (excitation/emission channels were set to 480/500 nm), and dead bacteria were stained red (excitation/emission channels were set to 490/635 nm). Each sample was examined using a 60 × objective lens in five randomly selected fields. The ratio of live bacteria in the biofilm was determined according to the coverage using Image Pro Plus 6.0 based on 10 random sights (Media Cybernetics, Inc., Silver Spring, MD).

### RNA isolation and real-time PCR (qRT-PCR)

To extract RNA and detect gene expression by qRT-PCR, 24 h biofilms were collected. TRIzol reagent (Invitrogen, Waltham, MA) was used to extract RNA and the PrimeScript™ RT Master Mix (Perfect Real Time) kit (Takara, Shiga, Japan) was used to synthesize cDNA. For qRT-PCR, each reaction mixture (20 μl) contained 2 × TB Green Premix Ex Taq II (10 μl), 50 × ROX Reference Dye (0.4 μl), cDNA (2 μl), forward and reverse gene-specific primers (0.8 μl each; specific primers are listed in Table 1) and nuclease-free water (6 μl) according to the handbook of the TB Green™ Premix Ex Taq™ II (Tli RNaseH Plus) kit (Takara, Japan). qRT-PCR was performed on the Step One Plus real-time PCR system (Applied Biosystems, Waltham, MA) as in our previous study [26]. Different gene expressions were normalized to the *16S rRNA* gene, and then analyzed by the 2^−ΔΔCT^ method.

**Table 1.** Primers used in this study

Primers	Nucleotide sequence (5′-3′)	References
*16S*-f	CCTACGGGAGGCAGCAGTAG	[25]
*16S*-r	CAACAGAGCTTTACGATCCGAAA	
*ldh*-f	AAAAACCAGGCGAAACTCGC	[25]
*ldh*-r	CTGAACGCGCATCAACATCA	
*gtfB*-f	AGCAATGCAGCCAATCTACAAAT	[25]
*gtfB*-r	ACGAACTTTGCCGTTATTGTCA	
*gtfC*-f	CTCAACCAACCGCCACTGTT	[25]
*gtfC*-r	GGTTTAACGTCAAAATTAGCTGTATTAGC	
*gtfD*-f	ACAGCAGACAGCAGCCAAGA	[25]
*gtfD*-r	ACTGGGTTTGCTGCGTTTG	
*atpD*-f	TGTTGATGGTCTGGGTGAAA	[25]
*atpD*-r	TTTGACGGTCTCCGATAACC	
*dexA*-f	TATTTTAGAGCAGGGCAATCG	[37]
*dexA*-r	AACCTCCAATAGCAGCATAAC	
*luxS*-f	ACTGTTCCCCTTTTGGCTGTC	[25]
*luxS*-r	AACTTGCTTTGATGACTGTGGC	
*brpA*-f	GGAGGAGCTGCATCAGGATTC	[25]
*brpA*-r	AACTCCAGCACATCCAGCAAG	
*comDE*-f	ACAATTCCTTGAGTTCCATCCAAG	[25]
*comDE*-r	TGGTCTGCTGCCTGTTGC	

### Cytotoxicity assays

L929 mouse fibroblasts were used to assess the cytotoxicity of SCH-79797 [27–29]. In brief, L929 cells were cultured at 37°C and 5% CO_2_ in a 96-well plate in DMEM containing 10% fetal bovine serum and allowed to attach overnight. The original medium was replaced with media containing 0.216, 0.108, 0.054, 0.027 μg/ml of SCH-79797, and 1% DMSO, and were cultured for 24 h. The Calcein-AM/PI double staining Kit (Dojindo, Kumamoto, Japan) was used to stain live and dead cells based on the manufacturer’s instructions. After staining for 30 min, cells were examined under a fluorescence microscope (Axio Vert. A1, Zeiss, Oberkochen, Germany). The survival rate of L929 cells under different concentrations of SCH-79797 was monitored by a Cell Counting Kit-8 (CCK-8) (Dojindo, Japan). In brief, after treated with SCH-79797 and 1% DMSO for 24 h, L929 cells were incubated with CCK-8 reagent for 1 h and OD values were measured at 450 nm (SpectraMax M5) and the survival rate was calculated using a blank control as reference.

### Statistical analyses

The experiments included at least three experimental samples in each group, and were repeated three times independently. One-way analysis of variance was performed to determine the significant effects of variables, and the SPSS 16.0 software (SPSS Inc., Chicago, IL) was used to perform a Tukey’s multiple comparison test.

## Results

### SCH-79797 inhibits biofilm formation of *S. mutans*

**Figure 1.**
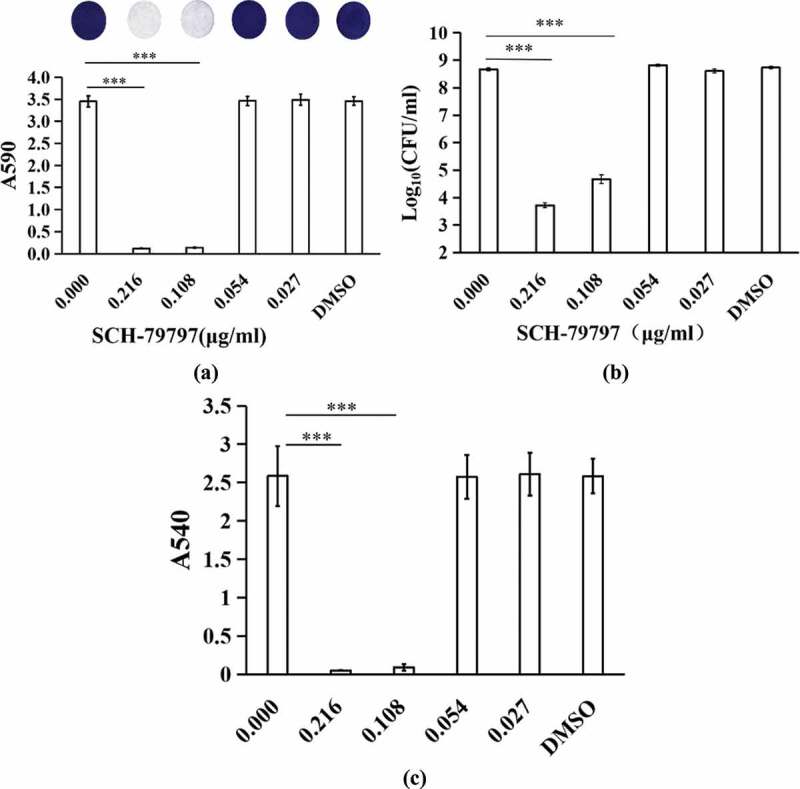
Biomass and vitality of biofilms. (**a**) Crystal violet staining; (**b**) CFU counts of *S. mutans* biofilms; (**c**) MTT assay of *S. mutans* biofilms. Data were expressed as mean ± standard deviation, *** means significant difference (*P < *0.005).

Results of the crystal violet staining demonstrated that when the concentration of SCH-79797 was 0.216 and 0.108 μg/ml, the biomass of *S. mutans* biofilm was remarkably inhibited (Figure 1(a), P *<* 0.005). When the concentration of SCH-79797 was 0.054 and 0.027 μg/ml, there was no significant difference in the OD value between these two groups and two control groups (*P >* 0.05). The DMSO group had no prominent influence on biofilm formation of *S. mutans* (Figure 1(a), P *>* 0.05). Similarly, the CFU results and metabolic activity of biofilms detected by the MTT assays count also indicated a similar trend (Figure 1b, c). When the SCH-79797 concentration was 0.216 and 0.108 μg/ml, the bacteria amount within biofilms and OD values of these two groups were significantly different from the control groups (*P <* 0.005). The presence of 0.054 and 0.027 μg/ml SCH-79797 and DMSO had no notetable effect on the metabolic activity of *S. mutans* biofilm (*P >* 0.05). In summary, SCH-79797 showed anti-biofilm effects and inhibited *S. mutans* biofilm formation.

### SCH-79797 obstructed EPS production of *S. mutans* within biofilms

**Figure 2.**
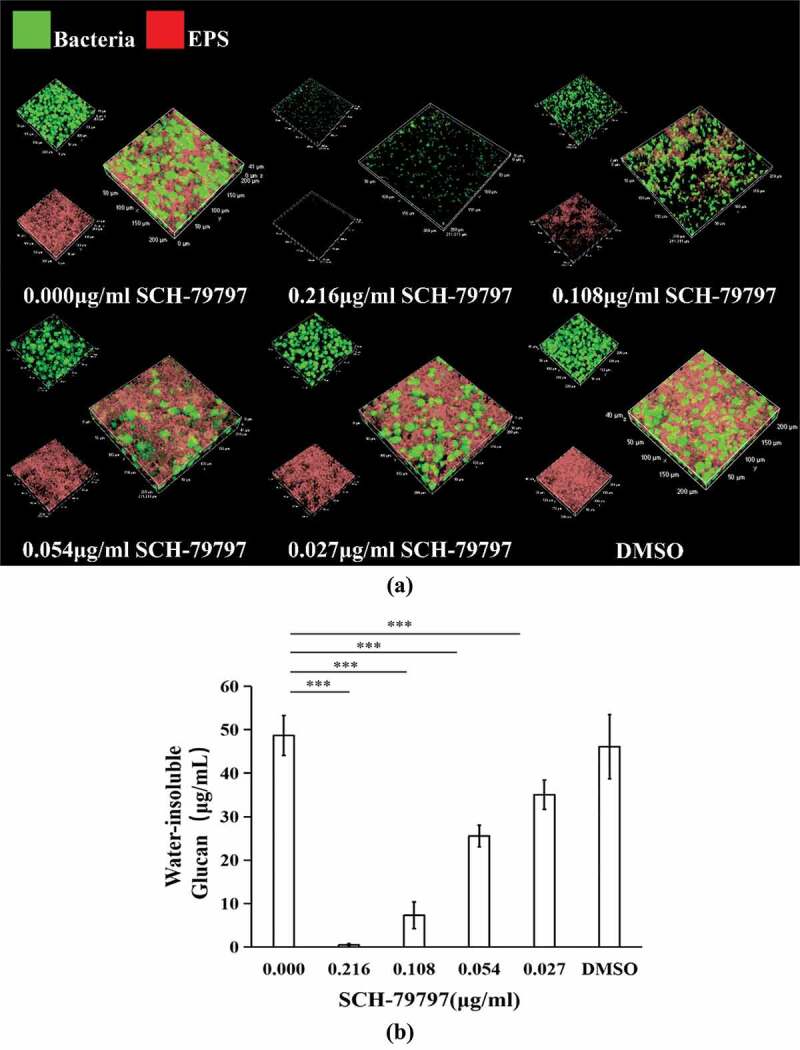
EPS production of biofilms. (**a**) EPS/ bacterial staining of *S. mutans* biofilm. Bacteria and EPS were stained green and red, respectively; (**b**) WIG of *S. mutans* biofilms generated at different concentrations of SCH-79797. Data were expressed as mean ± standard deviation, *** means significant difference (*P < *0.005).

After SCH-79797 was applied to *S. mutans* biofilm formation, qualitative EPS staining results showed the same trend as that of the quantitative result of WIG within biofilms. As shown in Figure 2(a), S. *mutans* was stained green, and EPS was stained red. As the concentration of SCH-79797 increased, *S. mutans* and EPS decreased in a dose-dependent manner. In quantitative results, when the concentration of SCH-79797 was 0.216, 0.108, 0.054, or 0.027 μg/ml, the biofilm WIG production was significantly lower than that of the blank control group (0.00 μg/ml SCH-79797), which decreased to 0.98%, 15.02%, 52.51% and 72.07% of the control group, respectively (Figure 2(b), P *<* 0.005). The WIG output of the DMSO group was not significantly different from that of the control group (P > 0.05).

### SCH-79797 suppresses acid production of *S. mutans* biofilms

**Figure 3.**
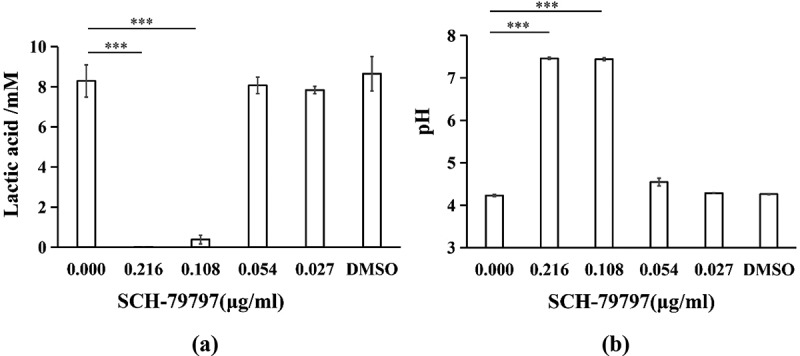
Acid production of biofilm. (**a**) Lactic acid production of *S. mutans* biofilm; (**b**) Supernatant pH of *S. mutans* cultured in BHIS for 24 h. Data were expressed as mean ± standard deviation, *** means significant difference (*P < *0.005).

As shown in Figure 3(a), the inhibitory effect of SCH-79797 on the lactic acid production of *S. mutans* in the biofilm was also dose-dependent. At 0.216 and 0.108 μg/ml groups, SCH-79797 hindered bacterial lactic acid production, which decreased to 0.19% and 4.63% of that observed in the control group respectively (*P <* 0.005) while the lactic acid production at 0.054, 0.027 and in the DMSO groups was not significantly influenced (*P >* 0.05). The supernatant pH of *S. mutans* biofilms also confirmed the suppressive effect of SCH-79797 on acid production. Similarly, when the concentrations of SCH-79797 were 0.216 and 0.108 μg/ml, the supernatant pH was about 7, which was significantly higher than that of the blank control group (Figure 3(b), P *<* 0.005). There was no significant difference in the supernatant pH of the remaining groups compared with the blank control group which all ranged from 4 to 5. This is lower than the critical pH of the dental enamel (*P >* 0.05).

### SCH-79797 restrains *S. mutans* biofilm formation by controlling bacterial growth

**Figure 4.**
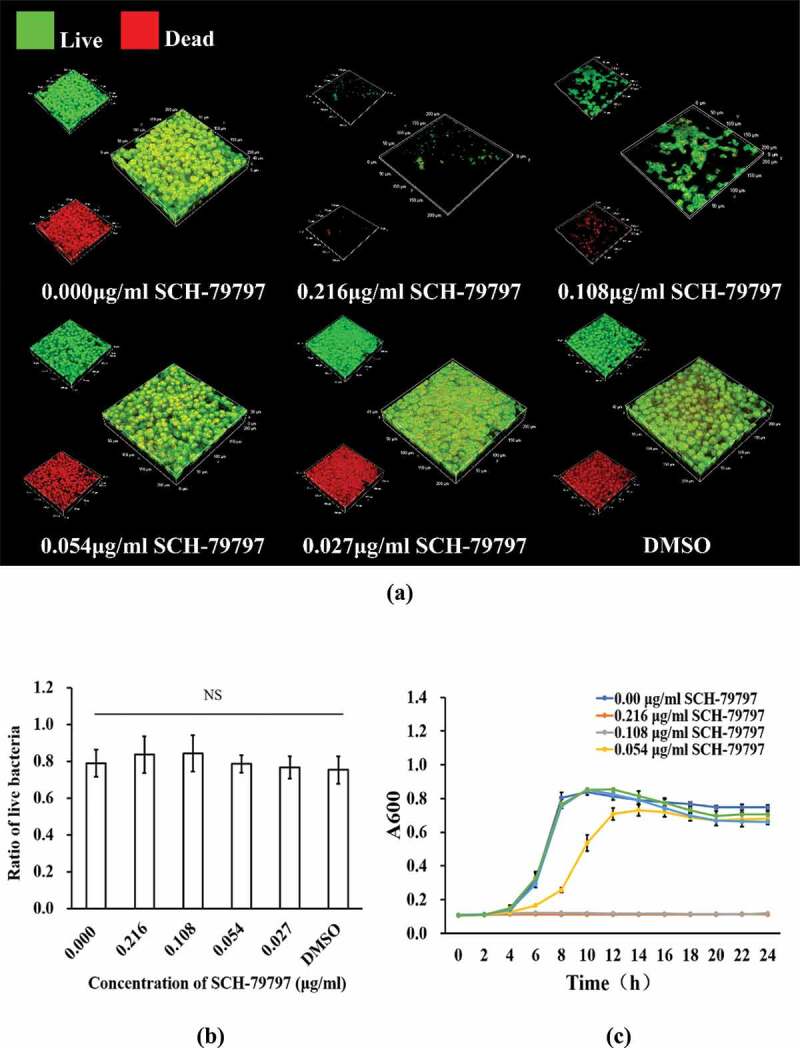
Bacteria live/dead staining and growth curve. (**a**) Live/ dead bacteria staining of *S. mutans* biofilm. Live bacteria were stained green and dead were stained red. Bar = 50 μm; (**b**) Ratio of live bacteria within biofilm according to live/dead bacteria staining results; (**c**) 24 h growth curve of *S. mutans* under different concentration of SCH-79797. Data were expressed as mean ± standard deviation, NS means no significant difference (*P > *0.05).

According to the bacterial live/dead staining results, both total live and dead bacteria in the 0.216 and 0.108 μg/ml groups were greatly reduced, while those of the remaining groups were not much different from each other (Figure 4(a)). At the same time, as shown in Figure 4(b), we calculated the ratio of live bacteria in each group of *S. mutans* biofilms. There was no significant difference among all groups. The 24 h growth curve of *S. mutans* showed that when SCH-79797 reached 0.216 and 0.108 μg/ml, the OD value of the two after 24 h hardly changed compared with that at 0 h. Although 0.054 μg/ml SCH-79797 delayed *S. mutans* growth before 12 h, there was no significant difference in the succeeding 12 h when compared to the blank control. By the data above, it could be concluded that SCH-79797 regulated bacterial biofilm formation by inhibiting the growth of planktonic *S. mutans*.

### SCH-79797 affects gene expression of *S. mutans* within biofilms

**Figure 5.**
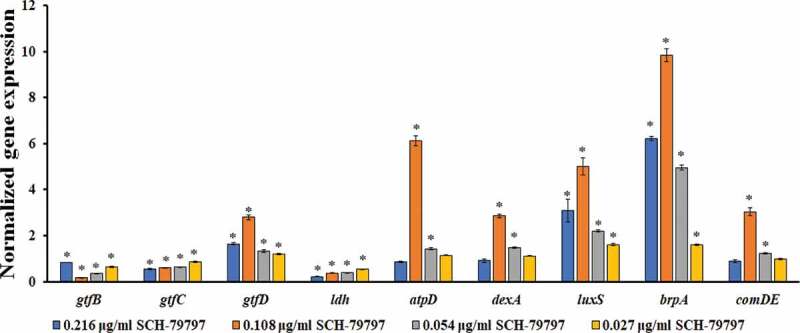
The gene expression of *S. mutans* under SCH-79797. mRNA expression levels of *gtfB, gtfC, gtfD, ldh, atpD, dexA, luxS, brpA* and *comDE* in *S. mutans* within biofilm. Data are expressed as mean ± standard deviation, * means significant difference (*P < *0.05).

Compared with that of the DMSO group, the expression levels of the bacterial biofilm polysaccharide-related genes *gtfB* and *gtfC* were significantly reduced (Figure 5, *P* *<* 0.05), while that of *gtfD* was significantly upregulated (Figure 5, *P <* 0.05). The expression level of *gtfB* in the 0.216 μg/ml group was higher than that of the other drug groups and still lower than that of the DMSO group (Figure 5). The *gtfC* expression decreased with the increase of the drug concentration, and the *gtfD* expression was the highest in the 0.108 μg/ml group (Figure 5). The acid production-related gene *ldh* of *S. mutans* was significantly down-regulated in each drug-containing group, and it was dose-dependent (Figure 5, *P <* 0.05). When the concentration of SCH-79797 was 0.108 and 0.054 μg/ml, the expression of the F1F0-ATPase β subunit gene *atpD* and the water-soluble glucanohydrolase gene *dexA* were significantly increased (Figure5, *P <* 0.05). The expression levels of *atpD* and *dexA* in the 0.108 μg/ml group were the highest, which were 6.1 and 2.8 times that of the DMSO group, respectively (Figure 5). The expression of the quorum sensing system-related genes *luxS, comDE*, and the effective biofilm formation and stress response involved gene *brpA* were significantly higher than those in the DMSO group, except for the *comDE* gene in the 0.216 and 0.027 μg/ml groups (Figure 5).

### SCH-79797 was essentially non-cytotoxic to L929 cells in the experimental concentration range

**Figure 6.**
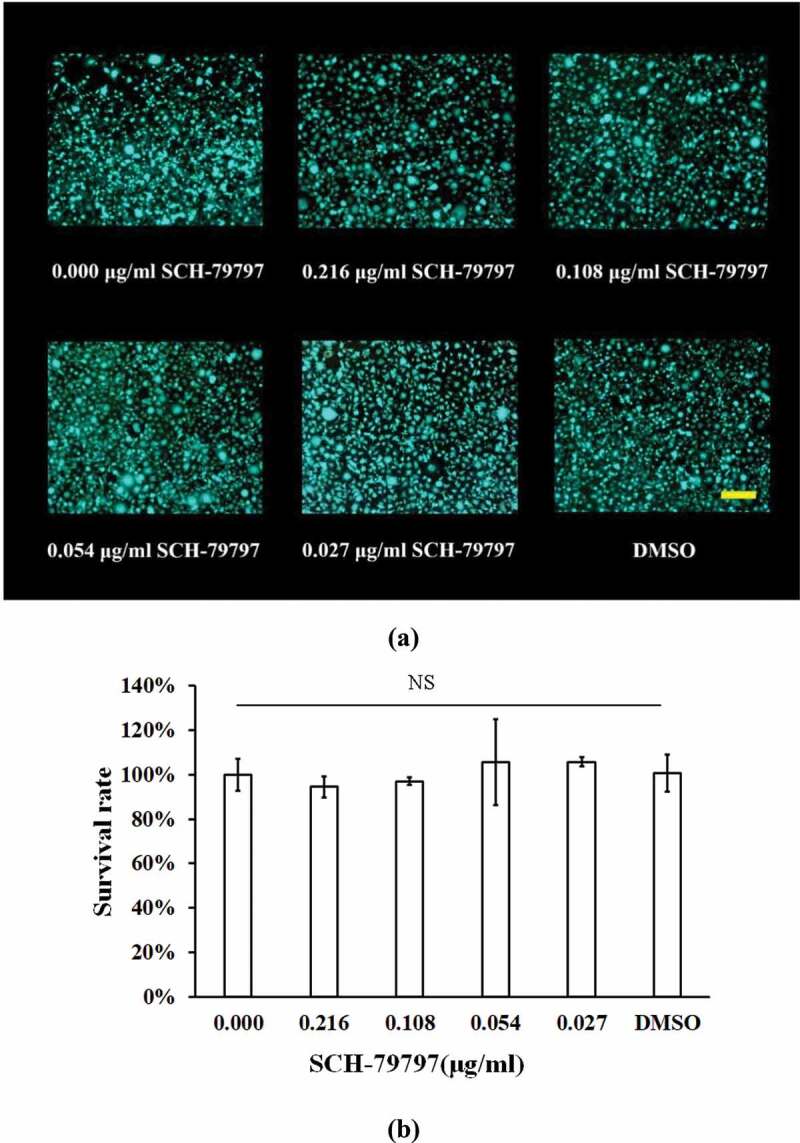
Cytotoxicity of SCH-7979 to L929 cell. (**a**) Live/dead images of L929 cells under the effect of SCH-79797. Live cells were stained green, dead cells were stained red. Bar = 100 μm; (**b**) Survival rate of L929 cell under SCH-79797 based on CCK-8. Data were expressed as mean ± standard deviation, NS means no significant difference (*P > *0.05).

To investigate the cytotoxicity of the experimental concentration of SCH-79797, live/dead cell staining and CCK-8 assays were employed. According to the live/dead images (Figure 6(a)), the number of L929 cells in the SCH-79797 and DMSO groups was not significantly altered compared with the blank control group and there was barely dead cell in all groups. Figure 6(b) shows that the survival rate of all groups were not considerably different from each other (*P >* 0.05).

## Discussion

In this study, we investigated the effects of SCH-79797 on *S. mutans* biofilms for the first time. We found that SCH-79797 showed an anti-biofilm outcome in addition to the bactericidal effect reported previously. SCH-79797 significantly inhibited *S. mutans* biofilm formation, acid production and EPS production. We also found that SCH-79797 could suppress *S. mutans* biofilm formation through bacterial growth inhibition rather than through killing bacteria. In addition, bacterial gene expression involving acid production and tolerance, polysaccharide synthesis and remodeling, biofilm formation and quorum sensing was influenced by SCH-79797. Finally, we verified that SCH-79797 had good biocompatibility to L929 cells within the experimental concentration range, revealing its anti-caries potential.

Acid produced by dental plaque is the key to demineralization of the tooth surface. Enamel is in the balance of demineralization and remineralization when the pH value is in the range of 5.0–5.5. When pH of the solution surrounding the enamel is lower than the critical value, demineralization is predominant over remineralization, resulting in cavity formation. In *S. mutans* acid is produced from glycolysis, in which lactate dehydrogenase encoded by *ldh* is the key enzyme [26,30]. We found that the 0.216 and 0.108 μg/ml SCH-79797 groups had considerably lower lactic acid production than other groups, and their pH was dramatically higher than the critical value. Meanwhile, the expression of *ldh* in each group containing SCH-79797 was significantly lower than that of the DMSO group (P < 0.05). Suppression of SCH-79797 on acid production of *S. mutans* biofilms resulted from both biofilm biomass reduction and repression of *ldh* expression This high supernatant pH and repressed acid production-related *ldh* expression under the effect of SCH-79797 implicated the prominently reduced demineralization ability of the biofilm, confirming the potential of this drug in caries control.

Gtfs secreted by *S. mutans*, imperative virulence factors in biofilm formation, can be adsorbed on the saliva membrane and microbial surface, allowing the conversion of sucrose into glucan [31]. In biofilms, the dextran-rich EPS matrix has various crucial functions, such as serving as a biofilm scaffold, adhesion and aggregation of bacteria on the surface, providing nutrients for bacteria, and reducing the efficacy of drugs on biofilms [32]. In this study, we found that SCH-79797 could restrain the EPS synthesis of *S. mutans* significantly. We further tested the expression of Gtfs-related genes (*gtfB, gtfC*, and *gtfD*). Among them, *gtfB* predominantly synthesizes α-1,3-WIGs, and *gtfC* mainly synthesizes a mixture of α-1,6-WSGs and WIGs, while *gtfD* synthesizes WSG3 [33]. It has been reported that GtfB and GtfC are highly homologous in amino acid sequences, and their coding genes could be expressed together and regulated by the same mechanism [34]. We found that the *gtfB* and *gtfC* expression in each SCH-79797 containing group was significantly downregulated when compared with that in the DMSO group. Combined with the biofilm biomass results, this might be used to explain why WIG in the SCH-79797 containing groups were prominently decreased. In the 0.216 μg/ml SCH-79797 group, *S. mutans* barely formed biofilm when compared with other groups, and its *gtfB* expression was also significantly suppressed while higher than in the other drug-containing groups. We speculated that although the suppression effect of *gtfB* expression still existed in this group, *S. mutans* tried to raise the *gtfB* expression to promote adhesion in response to the stress of SCH-79797. This hypothesis requires further study. Contrary to the expression trends of *gtfB* and *gtfC*, the *gtfD* expression (located upstream of the *gtfBC* site and it might be regulated in the opposite way to *gtfBC*) in each SCH-79797-containing group was significantly higher than that of the DMSO group [35]. Interestingly, *dexA*, which encodes DexA, is a water-soluble glucanohydrolase that could break down the α-1,6-glycosidic bond of WSG to provide energy for the bacteria, showed a similar expression tendency as that of *gtfD* [7]. The upregulated *dexA* and *gtfD* expression may result from the requirement of WSG hydrolysis to provide energy for the *S. mutans* under SCH-79797 stress.

*S. mutans* has developed a network of regulators that respond to environmental change in which quorum sensing plays an important role [36]. Quorum sensing enabled bacteria within biofilm to communicate further to organize a response within the population. In *S. mutans*, ComCDE mediates intra-species communication via competence stimulating peptide (CSP), and the LuxS system facilitates interspecies communication through autoinducer 2 (AI-2). It has been reported that *comD* (sensing CSP) and *come* (regulating the response of CSP) mutants would result in *S. mutans* biofilms with reduced biomass and a shortage of architectural integrity while *lusX* (responsible for AI-2 generation) mutation abates *S. mutans* biofilm formation. We found that the expression of *comDE* and *luxS* which were involved in the ComCDE and LuxS system respectively, were upregulated in response to SCH-79797. This might be attributed to bacteria tending to upregulate expression of these genes to further enhance biofilm formation against the anti-biofilm effect of SCH-79797.

xSCH-79797 was reported to exert a bactericidal effect by acting on bacterial folate metabolism and cell membrane integrity [15]. F1F0-ATPase, whose β subunit is encoded by *atpD*, is known as proton translocator and related to acid tolerance [26]. Its activity might be affected by SCH-79797 since it is located at the cell membrane resulting in upregulation of *atpD*. Analogously, upregulation of *brpA* might arise as a response to SCH-79797 targeting the cell envelope [37] since this gene is related to the integrity of the cell envelope [38,39]. In this study, SCH-79797 did not show thorough germicidal action but prevented *S. mutans* biofilm formation, which made it having potential to be a brand-new anti-caries agent without affecting the oral micro-ecological environment [40]. The actual biofilm inhibitory mechanism of SCH-79797 requires further investigation. SCH-79797 was reported as a dual-mechanism antibiotic to avoid drug resistance. Whether *S. mutans* would develop a corresponding resistance requires further investigation. Moreover, more complex biofilm models, such as multi-species and salivary biofilms, could be used to identify if SCH-79797 has potential to control biofilms in an ecological way against caries. Furthermore, as a positive potential to control caries, SCH-79797 could be used to modify dental materials such as composite resins and adhesives to endow them with anti-caries characteristics. Additionally, as a broad-spectrum antibiotic, the effectiveness of SCH-79797 on other biofilm-associated diseases in addition to caries is also worth exploring.

In summary, SCH-79797 displayed an anti-biofilm effect in this study, in addition to the bactericidal action uncovered formerly. Notably, it suppressed *S. mutans* biofilm formation and production of acid and EPS. Furthermore, SCH-79797 was proven to have good biocompatibility and could exert its anti-biofilm effect without damaging L929 cells. All the evidences in this study revealed its potential in caries control.
